# LncRNA EIF3J-AS1 functions as an oncogene by regulating MAFG to promote prostate cancer progression

**DOI:** 10.7150/jca.60676

**Published:** 2022-01-01

**Authors:** Chen Ye, Shengfei Qin, Fei Guo, Yue Yang, Huiqing Wang, Chao Zhang, Bo Yang

**Affiliations:** Department of Urology, Shanghai Changhai Hospital, P. R. China, Shanghai 200433, China

**Keywords:** Prostate Cancer, lncRNA, EIF3J-AS1, MAFG

## Abstract

Long non-coding RNAs (lncRNAs) can modulate various biological processes and behaviors in most human cancers. LncRNA EIF3J-AS1 has been reported as an oncogene in various tumors, but whether it exerts functions in malignant progression and gene expression in prostate cancer (PCa) remains unknown. In this study, we investigated the high level of EIF3J‐AS1 in PCa tissues and cells, and used functional assays to show that knocking down EIF3J‐AS1 inhibited PCa cell proliferation and metastatic ability. A preliminary mechanistic investigation also showed that EIF3J‐AS1 may increase the expression of MAF bZIP transcription Factor G (MAFG) in PCa. The expression correlation between EIF3J‐AS1 and MAFG was found to be positive in PCa tissues. Finally, rescue assays showed that MAFG might be involved in the EIF3J-AS1-mediated malignant phenotype in PCa cells. This study demonstrated that EIF3J-AS1/MAFG may play a key role in facilitating PCa progression.

## Introduction

Prostate cancer (PCa) is the most common malignant tumor and the second leading cause of cancer death among men worldwide 1. Despite many studies that aimed to improve the diagnosis and treatment of PCa, including PSA screening, PSMA theranostics, and new generation androgen deprivation therapy; the distant metastasis, castration resistance and neuroendocrine differentiation continue to limit long-term survival 2-5. Exploring the underlying molecular mechanisms of these processed from different perspectives may help us better understand the tumorigenesis and progression of PCa.

LncRNAs are a class of non-noncoding RNAs that are greater than lengths 200 nucleotides. Studies have shown that lncRNAs play important roles in cancer biological processes, such as proliferation, apoptosis and metastasis 6, 7. Recently, lncRNA EIF3J antisense RNA 1 (EIF3J‐AS1) was found to function as an oncogene in glioma, colorectal cancer, esophageal cancer, etc. 8-10, demonstrating its clinical potential as a biomarker and therapeutic target. However, the role of EIF3J-AS1 in PCa remains unclear. From the results of a past study, we demonstrated that EIF3J-AS1 is upregulated in PCa cells and tissues, and downregulation of EIF3J-AS1 inhibited cell growth and metastasis in PCa cells.

Previous research has demonstrated that lncRNAs can regulate gene expression through a variety of mechanisms, such as chromatin modification, transcriptional regulation and posttranscriptional regulation, by interacting with DNA, RNA and proteins 11-13. How EIF3J-AS1 regulates which key genes in PCa influence tumorigenesis and malignant progression in PCa must be studied. In the results of analysis a past study, we found that EIF3J-AS1 is positively correlated with MAFG (MAF bZIP transcription Factor G) in PCa cells and tissues by analyzing a public database and performing sample validation. MAFG is a bZIP-type transcriptional regulator that can bind to DNA as a homodimer, leading to transcriptional repression owing to a lack of transactivation domains 14. Previous studies have shown that MAFG contributes to the malignant progression of hepatocellular cancer and colorectal cancer 15, 16. In this study, we found that MAFG could promote cell proliferation and metastasis in PCa, and downregulating MAFG can impair the cancer-promoting effects of EIF3J-AS1 in PCa. These findings described the basics of the cancer-promoting role and regulatory mechanism of the EIF3J-AS1/MAFG axis in PCa progression.

## Materials and methods

### Tissues samples and cell lines

All 36 paired PCa and matched adjacent healthy control (HC) tissues were obtained from the urology department of Shanghai Changhai Hospital between 2016 and 2019. All patients provided consent for the use of all samples, and the collection of human tissue samples was approved and supervised by the Ethics Committee of Changhai Hospital.

### Cell culture and transfection

The human PCa cell lines PC-3, LNCaP, and DU-145 and the normal prostate epithelial cell line RWPE-1 were obtained from the American Type Culture Collection (ATCC). The PCa cell lines were cultured in RPMI 1640 medium supplemented with 10% fetal bovine serum (FBS, Gibco, USA). RWPE-1 cells were cultured in keratinocyte-SFM that was supplemented with epidermal growth factor and bovine pituitary extract (Gibco, USA). All cells were maintained at 37 °C in humidified air containing 5% CO2 according to the manufacturer's instructions. The siRNAs designed for EIF3J-AS1-AS1, MAFG and corresponding controls were all procured from GenePharma (Shanghai, China) for gene silencing. LV-NC and LV-MAFG for gene overexpression were also procured from GenePharma (Shanghai, China). Cell transfections were performed by using Lipofectamine 2000 (Invitrogen, U.S.A.) following to the manufacturer's instructions.

### Cell proliferation, migration and invasion assay

Cells were plated in 96-well plates (2000/well). After 24 h, 48 h, 72 h, and 96 h of incubation, cell viability was determined by Cell Counting Kit 8 assay (CCK8) with plate reader ELx800™ (BioTek, USA) according to the manufacturer's instructions. Transwell assays were performed to determine cell migration and invasion ability. Starvation-treated (serum-free for 24 h) cells were seeded (5×104) in the upper chamber of transwell inserts without or without Matrigel-coated membranes (Corning, USA) and cultured with serum-free medium. The lower chamber contained complete medium. After 48 h of incubation, the cells on the upper side of the membrane were scraped off, and the lower side cells were fixed with 4% paraformaldehyde and then stained with 0.1% crystal violet.

### RNA isolation and qRT-PCR

Total RNA was extracted from cells or tissues using TRIzol Reagent (Invitrogen, USA), and cDNA was synthesized using the PrimeScript™ RT Kit (TaKaRa, Japan) following the manufacturer's protocol. Quantitative RT-PCR was performed using SYBR® Premix Ex Taq™ (TaKaRa, Japan) in a StepOne-plus Real-Time PCR system (ABI, USA). The relative expression of EIF3J-AS1 and MAFG was calculated by the 2-ΔΔCt method.

### Western blot assay

All cells were collected and lysed in RIPA lysis buffer. Protein concentrations were detected by BCA assay (Thermo Scientific, USA) following the manufacturer's protocol. Proteins were separated by SDS/PAGE and transferred to PVDF membranes. Then, the membranes were incubated in blocking buffer for 1 h and incubated with primary antibody overnight. On the second day, the membrane was washed in PBS three times and incubated with secondary antibody for 1 h. The primary antibodies used were MAFG (Abcam, ab154318) and β-actin (Abcam, ab8226).

### Statistical analysis

Statistical analyses were performed with SPSS 17.0, and numerical data are shown as the mean ± SD of three independent experiments. Paired Student's t tests, one-way ANOVAs, and Pearson correlation analyses were performed for comparisons, and a pA P value (* p< 0.05; ** p< 0.01; *** p< 0.001) was considered significant.

## Results

### EIF3J-AS1 is upregulated in PCa tissues and cells

**Figure 1:**
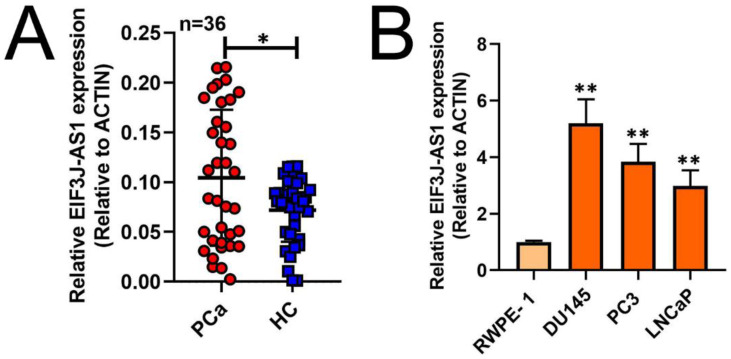
** EIF3J-AS1 is upregulated in PCa cells and tissues.** A and B, qRT-PCR assessed EIF3J-AS1 expression in PC tissues and control normal tissues, as well as in PC cells (DU145, PC3 and LNCaP) and the human normal prostate epithelial cell line RWPE-1. *P < 0.05, **P < 0.01, ***P < 0.001

To investigate the role of EIF3J-AS1 in PCa, we first measured the expression of EIF3J-AS1 in 36 paired PCa and matched adjacent HC tissues by qRT-PCR. The expression level of EIF3J-AS1 was significantly upregulated in PCa tissues compared with HC tissues (Figure 1A). Consistently, EIF3J-AS1 was upregulated in DU145, PC3 and LNCaP PCa cell lines compared with the normal prostate epithelial cell line RWPE-1 (Figure 1B). Considered together, these findings suggest that EIF3J-AS1 may play an important role in the progression of PCa.

### Downregulation of EIF3J-AS1 inhibits cell growth and invasion in PCa cells

**Figure 2:**
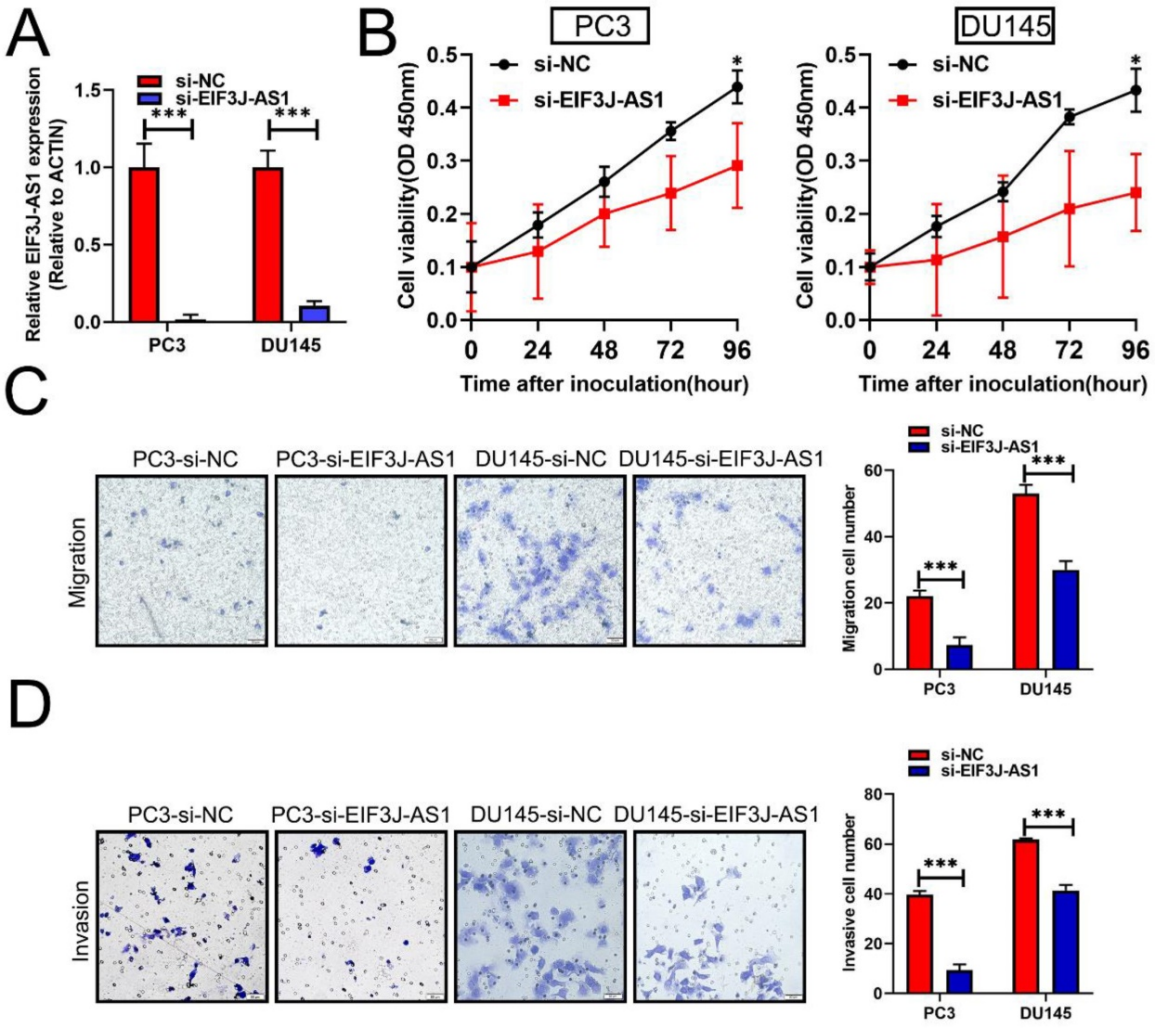
** Downregulation of EIF3J-AS1 inhibits cell growth and invasion in PCa cells.** A, qRT-PCR was used to assess EIF3J-AS1 expression in the indicated treated PC cell lines. B, The proliferation of si-EI3J-AS1-transfected cells was determined by CCK-8 assay. C and D, Transwell assays were performed to confirm the migration and invasion of the indicated PCa cell lines. *P < 0.05, **P < 0.01, ***P < 0.001

Based upon the above clinical data described above, we therefore performed several in vitro assays to describe the biological relevance of EIF3J-AS1 in PCa. Because PCa cells had higher levels of EIF3J-AS1, we treated these cells with EIF3J-AS1 knockdown (Figure 2A). In the CCK-8 assay, we observed that EIF3J-AS1 siRNA treatment markedly inhibited cell proliferation in PC3 and DU145 cells (Figure 2B). We also evaluated the invasion ability of control cells and EIF3J-AS1-knockdown PCa cells. In transwell migration and invasion assays, we observed that EIF3J-AS1 siRNA treatment markedly inhibited cell migration and invasion in PC3 and DU145 cells (Figure 2C and D). Collectively, results showed that EIF3J-AS1 contributes to cell growth and invasion in PCa cells.

### EIF3J-AS1 is positively correlated with MAFG in PCa

**Figure 3:**
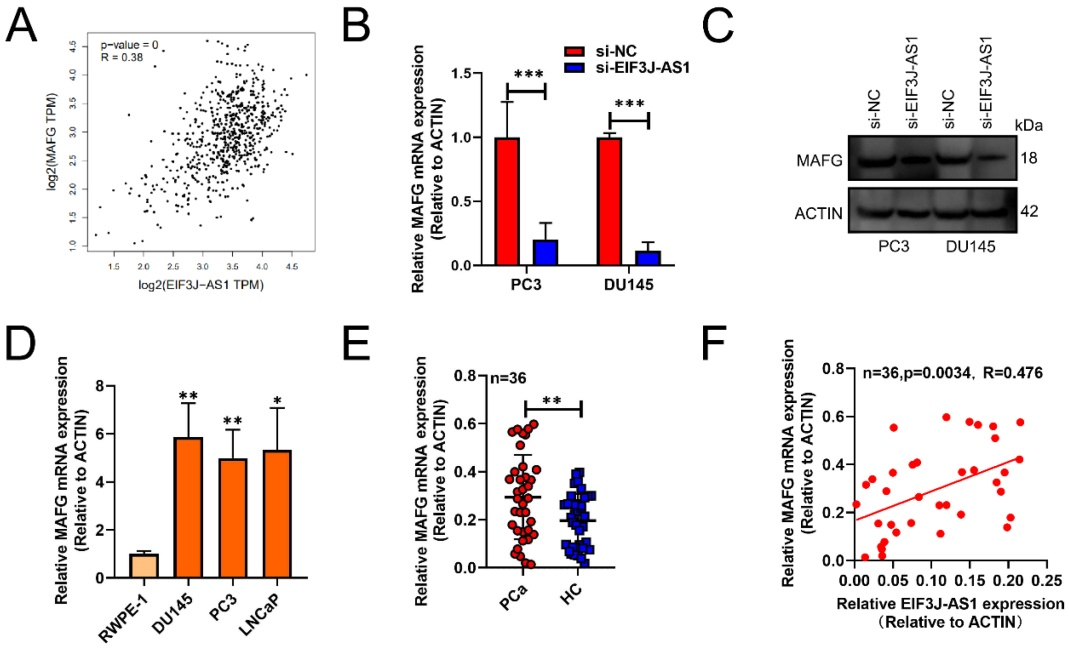
** EIF3J-AS1 is positively correlated with MAFG in PCa.** A, GEPIA assessed EIF3J-AS1, and MAFG was positively correlated in PCa tissues. B and C, MAFG mRNA and protein expression in PC-3 and DU145 cells transfected with si-EI3J-AS1 was evaluated by qRT-PCR and western blot assay. D, qRT-PCR was used to assess EIF3J-AS1 expression in PCa cells (DU145, PC3 and LNCaP) and the human normal prostate epithelial cell line RWPE-1. E, MAFG mRNA expression in clinical tissue was assessed by qRT-PCR. F, The correlation between MAFG expression and EI3J-AS1 expression in 36 PCa tissues was evaluated by Pearson's correlation analysis. *P < 0.05, **P < 0.01, ***P < 0.001.

Subsequently, we used GEPIA databases (http://gepia.cancer-pku.cn/) to screen out that EIF3J-AS1 was positively correlated with MAFG in PCa tissues (p<0.001, r=0.38) (Figure 3A). The mRNA and protein levels of MAFG also markedly decreased in PC3 and DU145 cells transected with sh-EIF3J-AS1 (Figure 3B and C). MAFG mRNA was also upregulated in DU145, PC3 and LNCaP PCa cell lines compared with the normal prostate epithelial cell line RWPE-1 (Figure 3D). We also verified the positive correlation of MAFD and EIF3J-AS1 at the mRNA level in 36 PCa tissues from Changhai Hospital Biobank. (Figure 3E-F). These results suggested that MAFG is upregulated in PCa tissues and cells and has a positive correlation with EIF3J-AS1.

### Knockdown of MAFG inhibits PCa cell proliferation and invasion in vitro

**Figure 4:**
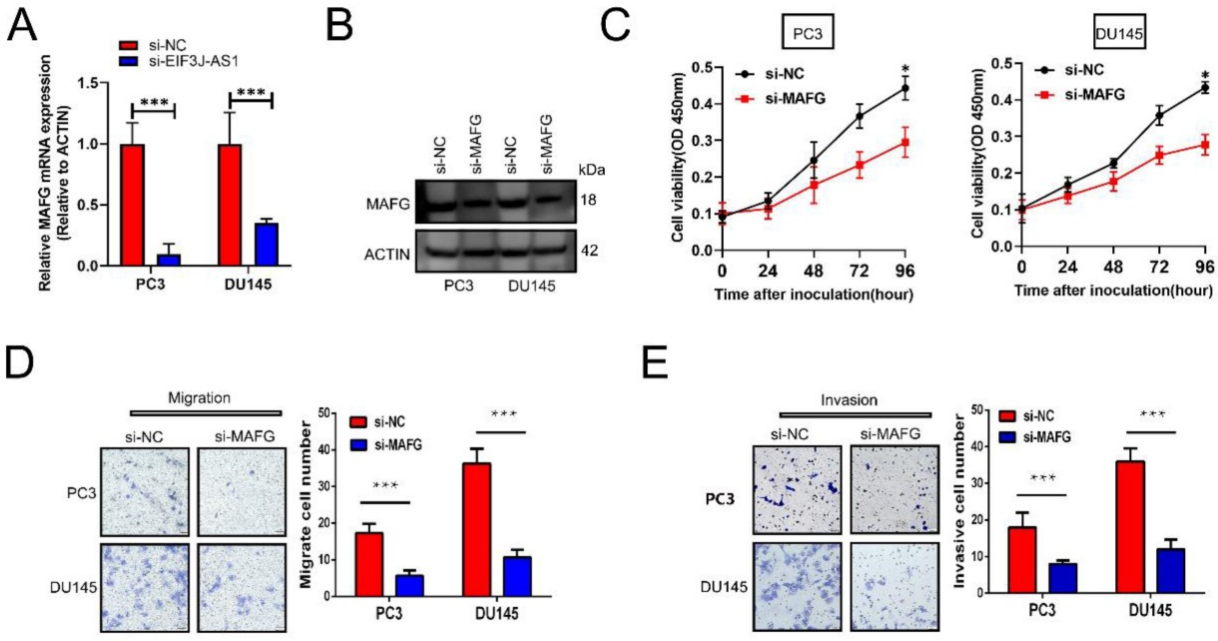
** Knockdown of MAFG inhibits PCa cell proliferation and invasion in vitro.** A and B, qRT-PCR and WB assays assessed MAFG mRNA and protein expression in the indicated treated PCa cell lines. C, The proliferation of the indicated PCa cells was determined by CCK-8 assay. D, The migration of the indicated PCa cells was determined by Transwell assay without Matrigel-coated membranes. E, The invasion of the indicated PCa cells was determined by Transwell assay with Matrigel-coated membranes. *P < 0.05, **P < 0.01, ***P < 0.001.

We then explored the effect of MAFG on the malignant phenotype of prostate cancer cells. First, appropriate cellular models were used with PC3 and DU145 cell lines because of their relatively high expression of MAFG. We treated those cells with MAFG knockdown (Figure 4A and B). The CCK-8 assay showed that knockdown of MAFG remarkedly inhibited the proliferation of both PC3 and DU145 cells (Figure 4C). Moreover, MAFG knockdown groups also indicated decreased migration and invasion ability in PC3 and DU145 cells (Figure 4D and E). Collectively, results showed that MAFG acts as an oncogene in PCa.

### EIF3J-AS1 enhances PCa progression by upregulating MAFG

**Figure 5:**
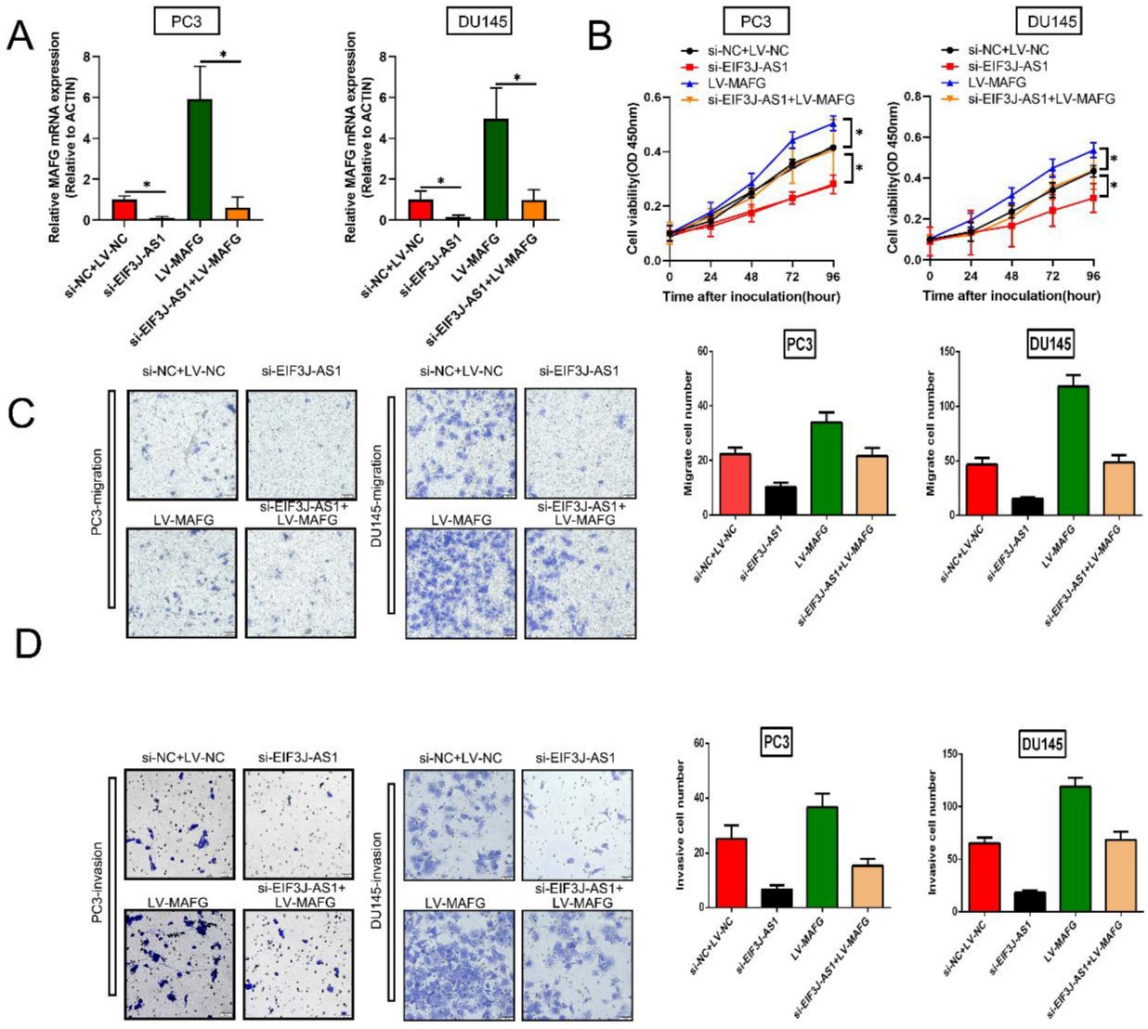
** EIF3J-AS1 enhances PCa progression by upregulating MAFG** A. qRT-PCR assays assessed MAFG mRNA expression in the indicated treated PC cell lines. B, Proliferation in the indicated PCa cells was determined by CCK-8 assay. C, The migration of the indicated PCa cells was determined by Transwell assay without Matrigel-coated membranes. D, The invasion of the indicated PCa cells was determined by Transwell assay with Matrigel-coated membranes. *P < 0.05, **P < 0.01, ***P < 0.001

Finally, we explored whether EIF3J-AS1 enhanced PCa cell growth by regulating MAFG by conducting rescue assays. We increased MAFG in PCa cells with EIF3J-AS1 knockdown (Figure 5A). The results of CCK-8 assays revealed that depleted EIF3J-AS1 markedly restrained the proliferation of PCa cells, but the transfection of LV-MAFG mitigated this effect (Figure 5B). In addition, increased MAFG countervailed the si-EIF3J-AS1-mediated alleviation of migration and invasion (Figure 5C and D). Thus, EIF3J-AS1 enhances PCa progression by upregulating MAFG.

## Discussion

In recent years, an increasing number of studies have shown that the dysregulation of lncRNAs, such as lncRNA MALAT1 and DRAIC, plays important roles in the initiation and development of PCa^17^-^20^. LncRNA EIF3J-AS1 was found to function as an oncogene in different kinds of cancer 8, 10 and to induce chemoresistance^21^. However, the role of EIF3J-AS1 has not been described with regard to PCa. In this study, we showed that EIF3J-AS1 was highly expressed in PCa tissues and cells, and knockdown of EIF3J-AS1 inhibited PCa cell proliferation, migration and invasion. These results described the driver of EIF3J-AS1 in PCa malignant progression. Thus, we further focused on how EIF3J-AS1 exerts its effects on PCa cells.

Previous studies showed that EIF3J-AS1 can exert its cancer-promoting effects by upregulating the expression of target genes, such as AKT1, YAP1 and ANXA11^8^-^10^, via competing endogenous RNA^22^, ^23^. In this study, according to bioinformatics analysis showed that EIF3J-AS1 and MAFG were positively correlated in PCa tissues. MAFG is a bZIP-type transcriptional regulator that belongs to the small MAF (sMAF) protein family 14, 24. Previous studies showed that MAFG contributed to the malignant progression of cholangiocarcinoma, colorectal cancers and chemotherapy resistance 15, 25, acting as an oncoprotein. Even in hepatocellular cancer, a study showed that MAFG mRNA levels are at least 2-fold higher than those in adjacent nontumorous tissues in approximately half of clinical specimens 15. However, the clinical significance and biological role of MAFG in PCa remain unknown. Further qRT-PCR experiments also validated that MAFG was significantly upregulated in PC tissues and cell lines. In addition, downregulation of MAFG in PCa cells resulted in the inhibition of proliferation and suppressed migration and invasion. In terms of preliminary mechanistic research, we found that downregulation of EIF3J-AS1 resulted in downregulated expression of MAFG in PCa cells. Rescue assays also showed that MAFG was involved in EIF3J-AS1-mediated PCa cell proliferation, migration and invasion. Thus, in this study, we provided evidence for a novel link between MAFG and EIF3J-AS1 in prostate cancer.

Thus, our study demonstrated that EIF3J-AS1/MAFG may play a key role in facilitating PCa progression and may serve as diagnostic biomarkers and potential therapeutic targets in PCa. However, the clear molecular mechanisms by which EIF3J-AS1 regulates MAFG upregulation and MAFG enhances cell growth and invasion in PCa must be further investigated in future research.
